# Vaginal delivery of triplets after emergency transvaginal cerclage: A case report and literature review

**DOI:** 10.1097/MD.0000000000037262

**Published:** 2024-03-15

**Authors:** Tong Zou, Yi-Cheng Wu, Qiang Yao

**Affiliations:** a West China Second Hospital of Sichuan University, Chengdu, China; b Key Laboratory of Birth Defects and Related Diseases of Women and Children (Sichuan University), Chengdu, China.

**Keywords:** case report, emergency transvaginal cerclage, preterm delivery, triplet

## Abstract

**Rationale:**

To report a peculiar case of vaginal delivery of a triplet after emergency transvaginal cerclage and to find a way to optimize some extreme situations encountered in clinical practice after evaluating feasibility.

**Patient concerns:**

A 33-year-old gravida 6, para 0050 woman at 21 + 6 weeks of gestation was referred to the obstetric department for opening of the cervical canal. An emergency McDonald cerclage was performed at 22 weeks of gestation after a comprehensive assessment, and the pregnancy ended with vaginal delivery at 24 + 6 weeks of gestation. The postpartum period was normal, and the newborns were discharged to home care after treatment in the neonatal intensive care unit.

**Interventions:**

After discussing the risks, the patient requested emergency transvaginal McDonald cerclage at 22 weeks of gestation.

**Outcomes:**

Emergency McDonald cerclage was performed at 22 weeks of gestation, and the pregnancy ended with vaginal delivery at 24 + 6/25 weeks of gestation, successfully prolonging gestation by 20/21 days. The postpartum period had no exceptional circumstances, and newborns were discharged to home care after treatment in the neonatal intensive care unit for 104/98/104 days.

**Lessons:**

Emergency cerclage seems to be impossible in multiple pregnancies. However, in this case, after a comprehensive assessment, it was feasible to extend the gestational age by emergency cerclage, and prompt and accurate evaluation is important to avoid complications and individualize the following management. In this case, we may find a way to optimize some extreme situations encountered in clinical practice and offer a glimmer of hope for families challenged with multiple pregnancies at risk of preterm delivery. However, more high-quality studies are needed to prove the effectiveness and safety of emergency cerclages in triplets.

## 1. Introduction

Multiple pregnancies increase the risk of miscarriage, stillbirth, preterm delivery, fetal growth restriction, and other complications, and the risk is even higher in triplets than in twins.^[1]^ The average gestational age of triplets is 31.9 weeks, and only 153.5 of every 100,000 births per year are triplets.^[2]^ With the median gestational age at delivery for triplets at 31.9 weeks, triplets are nearly 15 times more likely than singletons to die within 1 month of birth, and premature babies are at a significantly greater risk of incurring serious neonatal morbidities.^[3]^ Cervical dysfunction is an important factor in premature delivery, affecting 1% of pregnancies and 8% of recurrent miscarriages,^[4]^ usually screened using speculum or ultrasound. Regarding the treatment of cervical dysfunction, transvaginal cerclage is the most commonly used procedure to prolong gestation when preterm deliveries are about to occur. Emergency transvaginal cerclage was defined as a suture placed over the cervix with a premature cervical opening and membranes bulging into the vagina. However, cerclage remains a controversial issue in the prevention of preterm delivery in multiple pregnancies. Prophylactic cervical cerclage is not recommended in clinical guidelines for multiple pregnancies with neither medical history nor ultrasound indication,^[5–8]^ as studies have shown that there is no significant difference in either mortality or morbidity of neonatal incidence between twin pregnancies with or without prophylactic cervical cerclage.^[2]^ Only a few cases of gestation in triplets have been successfully prolonged by emergency cerclage.^[9–12]^ This paper reports a case of vaginal delivery after emergency cerclage of triplets and prolonged gestation for 20/21 days. After a 1-year follow-up, all 3 newborns were now in well-being.

## 2. Materials and methods

**Figure 1:**
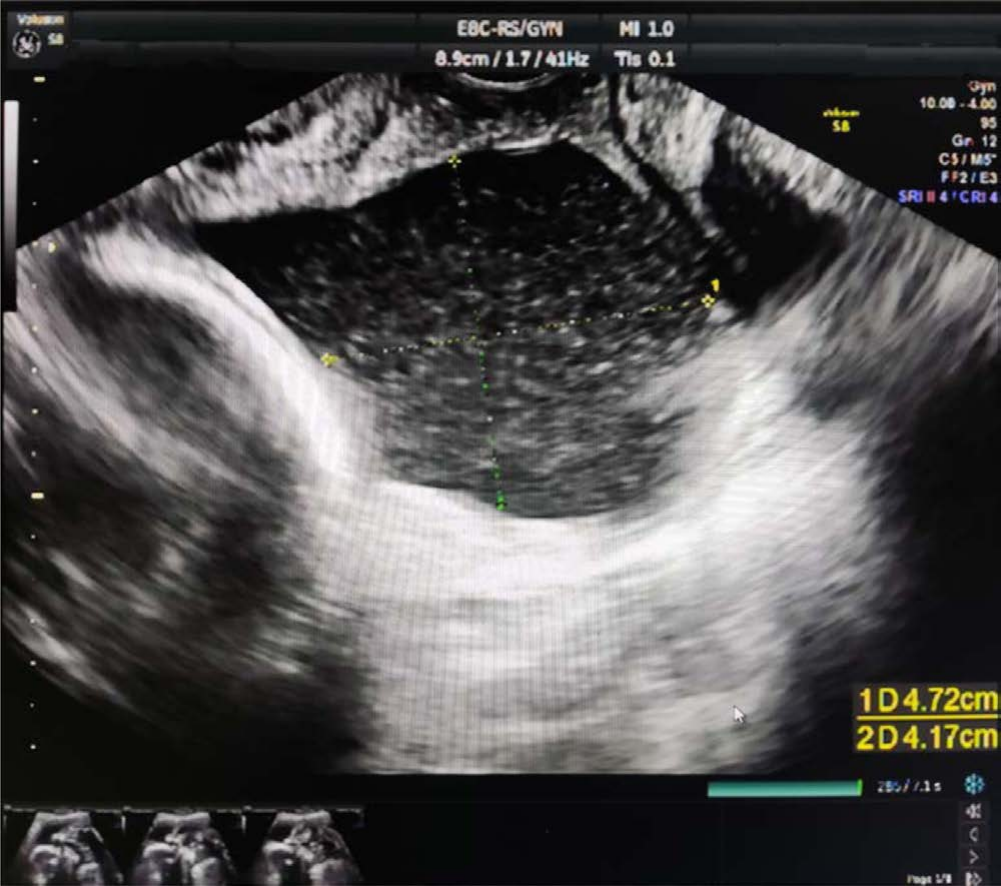
. TVS of cervix at 21 + 6 weeks of pregnancy. TVS = transvaginal sonography.

**Figure 2:**
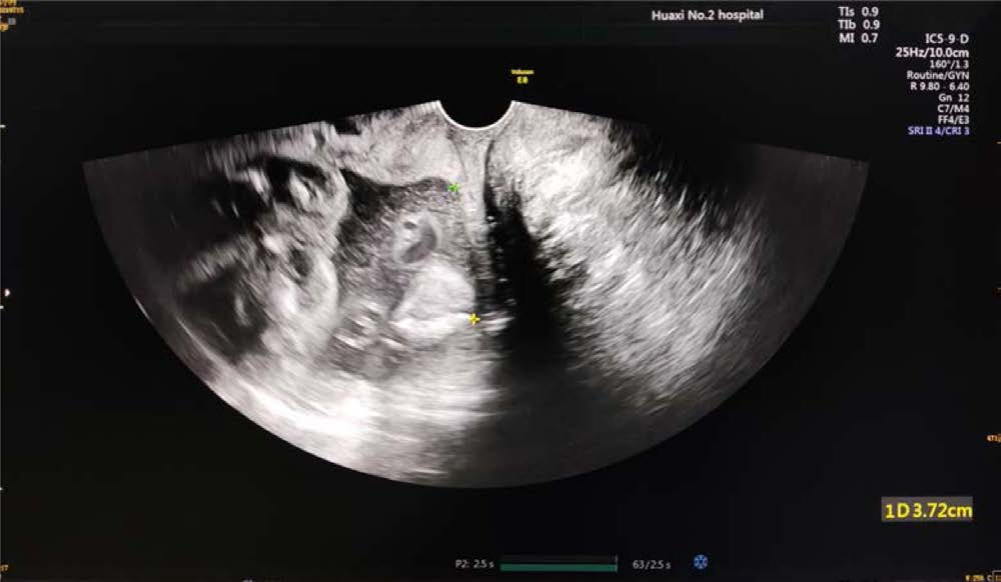
. Reopening of cervix at 24 + 5 weeks of pregnancy.

**Figure 3:**
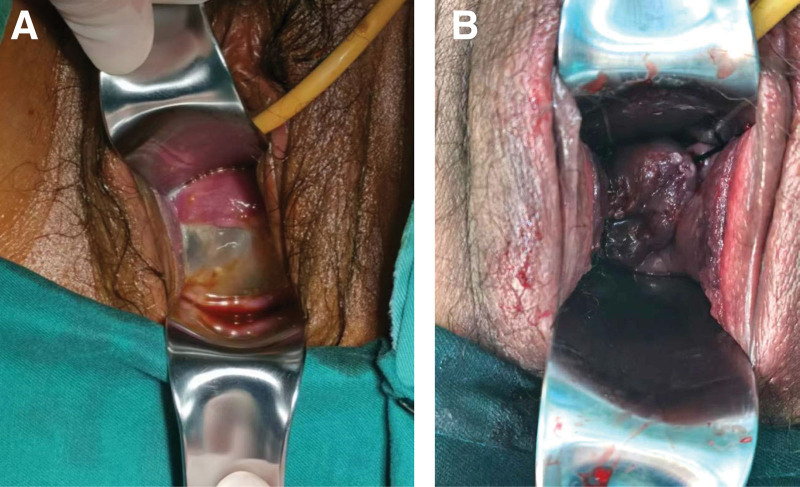
(A) The cervix before the cerclage. (B) The cervix after the cerclage.

A 33-year-old gravida 6, para 0050 woman at 21 + 6 weeks of gestation was referred to our obstetric department for opening of the cervical canal and was diagnosed with threatened miscarriage. The patient was pregnant using assisted reproductive technology, and 2 embryos were transferred 3 days after in vitro fertilization. Her previous history of pregnancy and birth included 1 tubal resection of ectopic pregnancy, 3 artificial abortions, and 1 embryo standstill. This primigravida had no cervical surgery, birth canal injury, or congenital reproductive tract malformation, and the only abnormality was multiple uterine fibroids, with the largest one being 3.1 cm in diameter. The first trimester ultrasound of the patient revealed dichorionic triamniotic gestation (Fig. 1) with 3 normal fetuses. Vaginal progesterone was administered due to intermittent vaginal bleeding during gestation. The patient had normal antenatal care until 21 + 6 weeks of gestation, when transvaginal sonography indicated shortening and opening of the cervical canal and, at the same time, an amniotic sac bulging into the vagina of 4.7 × 4.2 cm in size (Fig. 2); the patient was diagnosed with inevitable miscarriage. Sterile speculum examination showed an opening of the cervical orifice, which was dilated to 3 cm in size. Transvaginal sonography demonstrated that three fetuses had head or breech presentations with estimated fetal weights of 362 ± 53, 382 ± 56, and 362 ± 53 g. The patient’s initial laboratory examination on admission showed mild anemia (hemoglobin 101 g/dL) and a normal white blood cell count. The culture of the cervical secretions was positive for group B streptococcus and positive for *Ureaplasma urealyticum*. We informed the patient and her family of the controversies over cerclage and its poor prognosis in her case and ensured that they were fully aware that cerclage was not recommended in multiple pregnancies because existing research has shown that it could cause bleeding, abortion, premature rupture of membranes, and even premature delivery. In the end, they were asked for cerclage. Blood analysis and cervicovaginal swabs revealed no abnormal findings, and the patient was monitored for signs of clinical chorioamnionitis. Transvaginal cerclage was performed on the second day after comprehensive assessment, with the cervical orifice dilated to 3 cm and the amniotic capsule bulging into the vagina (Fig. 3A). A McDonald cerclage was performed, during which three 0.5 cm-long rubber tubes made of disposable catheters were added to the position of the sutures to prevent injury resulting from cutting pressure. The cerclage helped restore part of the cervical length to 2 cm with no funneling above the sutures, and the cervical orifice was closed (Fig. 3B). Postoperatively, uterine contraction inhibitors, such as ritodrine hydrochloride and atosiban, were used to inhibit uterine contraction. Complete blood cell and C-reactive protein tests indicated no signs of chorioamnionitis. During admission, infection detection was performed daily by taking the temperature and on a 2-day basis determination of white blood cell count and C-reactive protein, regular weekly reexamination of liver and kidney function, blood coagulation function, routine urination, and vaginal secretion culture. Serial ultrasound follow-up indicated normal development of triplet fetal growth and well-being. Daily ward rounds showed no abdominal pain, vaginal bleeding, elevated body temperature, or odor excretion. Oral iron supplements were used to correct the anemia, while antibiotics were used to treat the positive group B streptococcus and positive *U urealyticum*, and to prevent possible infections during the whole treatment. At 24 weeks of gestation, dexamethasone was used to promote the development and maturation of fetal lungs. Appropriate written consent for the publication of this case report was obtained from the patient.

## 3. Results

Unfortunately, at 24 + 6 weeks of gestation, due to the reopening of the cervical orifice and uncontrollable contractions, delivery was performed after removal of the cerclage thread and rubber tubes. Neonate A (female) in the breech presentation was delivered with Apgar scores of 6 and 9 at 1 and 5 minutes after delivery, and her birth weight was 650 g. Neonate B (male) in the occipital transverse position was turned into a breech presentation and was delivered with Apgar scores of 6 and 9 at 1 and 5 minutes after delivery, and his birth weight was 698 g. Neonate C (male) in the occipital transverse position was delivered with Apgar scores of 6 and 9 at 1 and 5 minutes, and his birth weight was 590 g. Chorioamniontic membrane swabs were obtained, and they were negative for bacteria. In the Neonatal Department, neonate A was diagnosed with respiratory distress (continuous positive airway pressure), pneumonia, and mild asphyxia; neonate B was diagnosed with neonatal early onset sepsis, respiratory failure (type I), respiratory distress, pneumonia, and mild asphyxia; neonate C was diagnosed with early onset septicemia of the newborn, respiratory failure (type II), respiratory distress, mild asphyxia, abnormal coagulation function, anemia, pathologic jaundice, brain injury in the premature infant, hyperglycemia, and respiratory acidosis. The triplets were discharged to home care after treatment in the neonatal intensive care unit for 104/98/104 days. After a 1-year follow-up, all 3 newborns were now in well-being, the mother’s postpartum condition is normal.

## 4. Discussion

Vaginal delivery of triplets after emergency transvaginal cerclage is extremely rare in clinical practice. Multiple intrauterine procedures may be a possible cause of cervical insufficiency in this patient in the early stages of pregnancy, and triplets also increase the possibility of preterm delivery. The surgical method is not difficult, but the assessment and decision to perform this procedure can be difficult and complex, and the stakes are high risk to the mother and fetus. Existing research has described only a few cases of delayed interval delivery after miscarriage of one fetus. Smith et al successfully prolonged triplet pregnancy gestation using robot-assisted prophylactic transabdominal cerclage (Table 1).

**Table 1:** Summary of the existing literature about cervical cerclage in triplet pregnancy.

Author(s)	Year; Number	Delivery interval (type)	Reason for treatment	Treatment (number)	Delivery mode (gestational age)	Outcome and conclusion
Banchi^[17]^	1984; 1	131(NA)	Miscarriage of one fetus at 16 GA.	TVC	CS (35 GA)	The remaining two infants survived.
Billion et al^[18]^	1991; 2	63(TTT) 14(DDT)	PPROM, prolapse of the cord, and miscarriage of one fetus at 21 GA.	extraction of the dead fetus at 21 GA, TVC	VD (30 GA) VD (23 GA)	Case 1: The remaining two infants survived. Case 2: Two weeks later the patient developed chorioamnionitis and septicemia, the remaining infants did not survive.
Van et al^[19]^	1991; 1	49(DTT)	PPROM and miscarriage of one fetus at 20 GA.	TVC, tocolysis, antibiotics	VD (27 GA)	The remaining two infants survived.
Chang et al^[20]^	1996;1	73(TTT)	Triplet pregnancy was reduced to twins at 14 GA, PPROM, and one fetus dead at 17 GA.	TVC, tocolysis, antibiotics, dead TVC, Fetus umbilical cord ligation,	VD (27 + 3 GA)	Only one infant survived.
Beinder et al^[21]^	1997;3	22(TTT) 70(DMT) 1(TTT)	Miscarriage of one fetus at 23 + 2 GA. Miscarriage of one fetus at 24 + 2 GA. Miscarriage of one fetus at 25 GA.	tocolysis, antibiotics	VD (26 + 3 GA) VD (34 + 2 GA) CS (25 + 1 GA)	Case 1: The remaining two infants survived. Case 2: The remaining infant survived. Case 3: The first fetus survived while the other two died, and the patient developed septicemia due to an infectious ovarian vein thrombosis. chorioamnionitis was the reason for a Caesarean section at 25 + 1 GA.
Trivedi et al^[22]^	1998;45	Triplet	NA	TVC, tocolysis, antibiotics	NA	The mean period of retention of the surviving retained twin/triplet was 48.9 ± 37.9 days compared to 25.7 ± 31.6 days for the dead retained twins/triplets (*P* = .08). The female-retained twins/triplets were retained much longer than the males (*P* = .008). The pregnancies lasted 45.9 days in the tocolytic group and 37 days in the nontocolytic group (*P* = .51). The delivery interval of the second born: 52 ± 42 days(TVC), 34 ± 30 days (no TVC) (*P* = .1). Tocolysis, cervical cerclage, and prophylactic use of antibiotics failed to make a statistically significant difference in the fetal outcome, and the birth weights, gestations, and sex of the retained twins/triplets affected their survival significantly.
Dieling et al^[23]^	1999;1	141(NA)	PPROM and prolapse of the cord at 18 + 2 GA, miscarriage of one fetus at 18 + 4 GA	TVC, tocolytic, antibiotics, bedrest	VD (38 GA)	The remaining two infants survived.
Biard et al^[9]^	2000;3	63(TTT) 44(TTT) 22(TTT)	Case 1: PPROM at 19 GA and prolapse of the cord at 21 GA leading to the miscarriage of one fetus; Case 2: miscarriage of one fetus at 24 GA; Case 3: preterm labor began with a 7 cm cervical dilation at 23 + 5 GA and miscarriage of one fetus.	PTVC and TVC (case 1), TVC (case 2/3), tocolytic, antibiotics, corticosteroids	CS (30 GA) CS (29 + 6 GA) CS (26 + 5 GA)	Case 1: the remaining two infants survived. Case 2: the remaining two infants survived. Case 3: an intrauterine death of the second triplet at 26 + 5 GA, only the third triplet survived. No maternal complications with sequelae are reported.
Rebarber et al^[24]^	2005;248	Triplet	Without cervical insufficiency history	PTVC	NA	No significant differences were seen in mean gestational age at delivery, incidence of preterm birth before 32 weeks, birth weight, or neonatal days in the hospital. PTVC did not improve pregnancy or neonatal outcomes in triplet pregnancies without a history of cervical insufficiency.
Bernasko et al^[25]^	2006;95	Triplet	Triplet	PTVC (55) and no PTVC (40)	NA	PTVC was not associated with a significant prolongation of triplet pregnancy.
Moragianni et al^[26]^	2009;24	Triplet	Ultrasound-indicated cervical shortening	TVC (11) and no TVC (13)	NA	Triplet pregnancies complicated by cervical shortening diagnosed on biweekly TVS surveillance do not appear to benefit front placement of cerclage, based on an assessment of gestational age at delivery, birth weight, and incidence of very low birth weight infants.
Moragianni et al^[27]^	2011;24	Triplet	Ultrasound-indicated cervical shortening	TVC (11) and no TVC (13)	NA	In triplet pregnancies, transvaginal ultrasound ascertainment of a shortened cervix at between 11 + 1 and 28 + 3 weeks gestation is associated with an increased risk of preterm delivery and poorer neonatal outcome. And these patients do not benefit from ultrasound-indicated TVC and can instead be safely managed expectantly.
Sumners et al^[12]^	2011;141	Triplet	Triplet (49); Non-classical indications and uterine anomaly^[4]^; Non-classical Indications^[7]^; Deep cervical laceration without Cervical incompetence history^[1]^; uterine anomaly^[4]^; Shortened cervix.^[5]^	PTAC (60); PTVC (31,2 failed and had rescue TVC/TAC); no TVC (50, 5 had rescue TVC).	NA	Prophylactic TAC was associated with reduced incidence of extreme prematurity and improved incidence of neonatal/postnatal survival. PTAC appears to lower the incidence of delivery before 28 weeks.
Wooldridge et al^[11]^	2012;1	28(TTT)	Miscarriage of one fetus at 17 GA.	Rescue TVC, antibiotics, corticosteroids, and daily progesterone.	VD (31 GA, forceps)	The remaining two infants survived but required ventilatory support for 72 h.
Young et al^[28]^	2014;24	Triplet	Cervical shortening.	TVC (16); no TVC (8)	Median GA: 31.3 (TVC), 29.8 (no TVC)	No benefit in terms of pregnancy prolongation or neonatal outcomes with TVC for triplet gestations complicated by an asymptomatic short cervix.
Smith et al^[29]^	2019;1	98(TTT)	History of cervical insufficiency and a failed TVC.	Robotic-assisted PTAC at 11 GW.	CS (35 + 2 GA)	All three infants survived and were healthy. Prophylactic robotic-assisted transabdominal cerclage by an experienced surgeon is a feasible alternative for patients with cervical insufficiency in higher-order multifetal gestation pregnancies.
Hassani et al^[30]^	2020;1	70(DTT)	PPROM at 16 GA and miscarriage of one fetus at 22 GA.	TVC, tocolysis, antibiotics	CS (30 GA)	The remaining third infants survived and were discharged from the hospital after 4 weeks in the neonatal department.
Bettencourt-Silva et al^[1]^	2021;1	58(DTT)	Ultrasound-indicated.	TVC, tocolysis, antibiotics, corticosteroids, magnesium sulfate	CS (28 GA)	All three infants survived. A case-by-case evaluation based on cervical shortening progression and a conscious parents’ decision seems to be a reasonable approach.

CS = cesarean section, DDT = diamniotic dichorionic twins, DMT = diamniotic monochorionic twins, DTT = diamniotic trichorionic triplets, GA = gestation age, PPROM = preterm premature rupture of membranes, PTAC = prophylactic transabdominal cerclage, PTVC = prophylactic transvaginal cerclage, TAC = transabdominal cerclage, TTT = triamniotic trichorionic triplets, TVC = transvaginal cerclage, VD = vaginal delivery.

Transvaginal cerclage is a commonly performed intervention in the care of women at risk of preterm birth and second trimester fetal loss.^[13]^ Nevertheless, its use, especially in emergency cases, has remained controversial for multiple pregnancies, where such risks increase. For women with cervical dilatation and fetal membrane exposure, emergency transvaginal cerclage is an alternative option, which can be performed after excluding premature rupture of membranes. This emergency procedure aims to prolong the duration of pregnancy^[14]^ and should be considered in twins where the cervix is dilated (>1 cm) before viability.^[8]^ It can reduce the rate of preterm birth in patients with painless cervical dilatation and protrusion of the fetal membranes, prolong gestational age, and improve newborn survival.^[14]^ In a meta-analysis of singleton pregnancies, 38 studies were reviewed regarding emergency transvaginal cerclage, and 12 observational analyses involving 1021 patients^[15]^ showed that emergency cerclage in pregnant women with painless cervical dilatation seems to decrease preterm births, prolong pregnancy, and decrease neonatal deaths and fetal losses, but does not increase the risk of chorioamnionitis or premature rupture of membranes. Nevertheless, these favorable results were associated with low-quality statistical analyses,^[15]^ and the study included only singleton pregnancies. In addition, an emergency transvaginal suture may also increase the risk of infection due to the exposure of the membranes to vaginal bacteria. However, studies have shown that the risk of chorioamnionitis and premature rupture of membranes during or after cerclage is similar to that associated with conservative management.^[16]^ Therefore, the effectiveness and safety of emergency cerclages remain controversial.

Despite only a few favorable evaluations of emergency transvaginal cerclage in multiple pregnancies, the existing literature includes only a very limited number of high-quality randomized controlled trials. Studies on triplets with cervical insufficiency are even fewer, and treatment recommendations based on large amounts of clinical evidence are lacking. Consequently, emergency cerclage seems to be mission-impossible or even forbidden. In the present case of a triplet with cervical insufficiency, however, we showed that it is feasible to extend the gestational age by emergency transvaginal cerclage. By doing so, we may find a way to optimize extreme situations encountered in clinical practice. Therefore, more high-quality studies are needed to prove the effectiveness and safety of emergency cerclages in triplets. Most importantly, we need to consider the wishes of the family, the patient’s pregnancy and childbirth history, their financial capacity, and the long-term health follow-up issues that may arise in newborns. Only by comprehensively considering the above issues can we make a decision that benefits the patient’s family more than harms them. We understand that a case alone is far from sufficient and that more evidence is needed to assess the safety, efficacy, and acceptability of emergency transvaginal cerclage in multiple pregnancies. However, we also believe that emergency transvaginal cerclage might also improve pregnancy and neonatal outcomes if first assessed comprehensively and then performed by experienced obstetricians. At best, it may offer a glimmer of hope for families challenged with multiple pregnancies at risk of preterm delivery.

## Author contributions

**Conceptualization:** Tong Zou, Qiang Yao.

**Data curation:** Tong Zou.

**Writing – original draft:** Tong Zou.

**Writing – review & editing:** Yi-Cheng Wu, Qiang Yao.
